# Multiteaching style and active reflection for swimming

**DOI:** 10.3389/fspor.2025.1679433

**Published:** 2025-10-13

**Authors:** Gabriele Signorini, Raffaele Scurati, Damiano Formenti, Athos Trecroci, Giampiero Merati, Roberto Del Bianco, Marta Rigon, Pietro Luigi Invernizzi

**Affiliations:** ^1^ Department of Biomedical Sciences for Health, Università degli Studi di Milano, Milan, Italy; ^2^ Department of Biotechnology and Life Sciences, Università degli Studi dell’Insubria, Varese, Italy; ^3^ IRCCS Fondazione don Carlo Gnocchi, Milan, Italy; ^4^ Federazione Italiana Nuoto, Rome, Italy; ^5^ UCAM Catholic University of Murcia, Murcia, Spain

**Keywords:** perceived aquatic competence, actual motor competence, systems thinking, methodological competence, didactical competence, instructors’ training, psychosocial skills, dose–response

## Abstract

A student-centred approach is widely used in team sports, but less so in swimming. A proper dosage of stimuli and a multiteaching approach, where games can link understanding and motor competence, could lead to educational success. This study investigated the didactical, methodological, and personal competencies of swimming instructors (SI) and their relationships with children's actual and perceived aquatic competencies. Two hundred children and 44 SI participated in the study. The Teaching Styles Questionnaire (TSQ) assessed the instructors' self-reported awareness of the teaching styles they use, which was compared with the experimenters' observations (as recorded by IESPES, System for Observing Fitness Instruction Time, and Instrument for Identifying Teaching Styles tools). The instructors' empathy and self-control were further evaluated, while two pictorial scales assessed the children's actual and perceived aquatic competence. TSQ confirmed the instructors' predominant use of monoteaching pedagogy, primarily characterized by linear (command and practice) styles (*p* < 0.001; *W* = 0.71). Even if SI exhibited general positive personal skills (empathy and self-control), a discrepancy between children's actual and perceived aquatic competence was found (*p* < 0.001; *r* = −0.83), with the latter overestimating the former. Conversely, the multiteaching approach of instructors directly correlated with didactic effectiveness (*r* = 0.64), empathy (*r* = 0.75), and children's actual (*r* = 0.63) and perceived aquatic competence (*r* = 0.65), suggesting that a multiteaching approach should also be used in swimming.

## 1 Introduction

Differently than in team sports, where learner-centred methodological approaches (contemporary learning) are widely used (1–3), and while a variety of methods are reported to be employed in teaching swimming by a recent review by Minkels et al. (4), learn-to-swim programs are generally characterized by teacher-centred conducts, typically implying elevated movement repetitions and emphasis on technical elements to be reproduced most stably and predictably (traditional learning) (5). Practice and reciprocal teaching styles are the prevalently used teaching approaches (5, 6). Thus, for a more comprehensive learn-to-swim approach, research should concentrate on the data collection and analyses of instructors' methodological approaches, the effects of teaching procedures on learners, and the benefits on the learners' skills development, even if not strictly connected to swimming, such as cognitive and psychosocial features. In other words, research should approach the concern under a systems thinking vision (7, 8).

Some theoretical models are more suitable for addressing the contemporary methodological approach to teaching swimming and deserve to be explained: the educational systems thinking (8), the constructivist approach to teaching (9), non-linear pedagogy (10), *teaching games for understanding (TGfU)* (11, 12), the sports education model (13), and competency models for sports instructors (14).

Systems thinking represents an idea and a methodological approach that encourages us to consider the set of elements that characterize a phenomenon. These elements represent “dynamic actors” composed of people, organizations, and norms in close and reciprocal relationship with one another and with the sociocultural context in which they are embedded. Socio-ecological currents consider different levels of systems (micro, meso, exo, and macro-systems), which, interacting with each other, form the basis of human development (15–17).

The constructivist approach aims to build a thought based on reflective practices through an exploratory-experiential path, incorporating situational and environmental awareness, to enhance educational training processes and products, considering the diverse contexts and variables of learning (18). It is linked to systems thinking through a thought based on relativism, complex systems of interactions, and non-linearity of interactions. The brain itself works in this mode: the neurons of the brain, directly involved in the processes of learning construction, highlight this concept of a complex constructivist system formed by many units that communicate with each other and exchange information, both incoming and outgoing, in an ever-changing way (non-linear modality) depending on the variety of stimuli that interact (19).

The relativistic conception of reality is based on the thought that everything depends on an infinity of factors (20). So, if in a positivist scientific interpretation, the “there is” represents the emblem of its conception, the “could be” represents the emblem of the relativistic conception. Constructivist epistemology does not pursue absolute truth and considers the interaction and connection between elements as the basis that composes the socio-ecological reality and that builds it together (21). Constructivist epistemology has also given rise to the emergence of complexity as a new perspective on reality and on how man knows (22).

The non-linear pedagogy approach is based on ecological theories, dynamic systems, and the self-organizing capacities of the organism, which determine a complex perspective.

The primary process that occurs between the components of a non-linear approach is feedback or retroactions: a component of the system acts directly or indirectly on another component, which in turn acts on the first component, creating a system complexity with a set of multiple different parts that self-organize and interact with each other (23).

Feedback can be both positive and negative. With negative feedback, the system achieves stability, as the second component inhibits the first, allowing the system to reach a dynamic equilibrium. With positive feedback, the system moves away from dynamic equilibrium as the different components of the system stimulate each other. The system is, therefore, not only complex but also continuously dynamic. To better understand these concepts, in a tank with an upper hole that permits the insertion of water and a lower hole that allows it to empty, if we insert water inside, leaving the lower hole open (negative feedback), the system “tank” will remain partially filled at the same water level (dynamic equilibrium). If we close the lower hole (positive feedback), the tank will fill, and it will be necessary to add another tank to continue to stream water (disequilibrium).

The complex perspective is adopted by infant research, which conceives the human being as a system characterized by internal self-organizing capacities, openness to the environment, and processes of self-regulation and interactive regulation with the environment (24). In this approach, the child is stimulated to acquire motor competence through changing circumstances constituted by environmental, organism, and task constraints and affordances, deliberately created through the programming of proposals designed to encourage the exploration of different motor solutions and the organism's self-organization capabilities.

One expression of the non-linear approach is the *teaching of games for understanding* (11, 25). In TGfU, the game represents the central idea of this methodology, which integrates understanding and mastering game strategies with awareness of the environment and the factors that influence it. The child learns to interpret different game situations and makes more effective decisions. This methodology is characterized by collaborations, discussions, and the continuous comparison implemented through the subject's relationships with the group of peers and with expert subjects (instructors) (26, 27).

The sports education model represents the educational direction that guides the application criteria of the previous models. This methodology is oriented towards a humanizing and psychosocial conception of sport, aimed not only at sporting success but, above all, at educational success within the context of sports education's goal of learning personal competences (13). An adequate dosage of quantity and quality of stimuli, involvement, inclusion, fair play, and the use of sport as a tool for adequate self-knowledge and self-awareness represent the key elements of this framework. This approach represents a model that, in the face of contextual diversification and obstacles connected to the complexity and non-linearity of educational processes, can contribute to the diffusion of physical literacy, well-being, and overall growth of the subjects (28, 29). In this context, the role of the teacher extends beyond teaching sports techniques. The teacher becomes a facilitator of learning, guiding students towards the development of motor, cognitive, and socio-affective skills. The instructors' competence model in sports refers to the model of a competent instructor and the components necessary for their role as sports instructors within the complexity of educational processes. These components can differ and have varying accents depending on the sociocultural context and the community in which the instructor carries out their activity (14). Understanding which components are most relevant in different contexts and how to identify them implies a comprehensive analysis of the instructors' abilities, skills, and attitudes, but also of the effects and effectiveness of these components on the psychomotor competence of the children they teach in a specific reality (30). The teacher/instructor's ability to choose among different teaching methods the most adequate for the learners facilitates the learning process and enhances their ability (31).

Nevertheless, in the teaching process, it cannot be sufficient to consider only the didactical/methodological competencies. Recent studies have shown that dynamic teacher-student interaction influences learning, and it comprises not only the teacher's ability to manage lessons didactically but also the ability to efficiently dialogue with learners to understand their needs and consequently adapt the lesson and their own behavior. Hence, the instructor's empathy and self-control become highly important and determinant for the acquisition of learners' aquatic competencies (32).

Scientific evidence in this field has given particular importance to the pedagogical and methodological areas, without neglecting empathy and communication skills as key elements of “an art of teaching” that must produce not only a competent motor athlete but also a positive perception of this competence with psychosocial implications (33). In this model, conscious passage through laboratory and experiential training gradually allows the acquisition of an intuitive competence capable of integrating the different information relating to the educational process by applying the most effective and diverse didactic-methodological solutions, considering the specific contextual characteristics (34). The game, the exercise, and the procedures, even if effective, “are not the teacher” and cannot be considered as exclusive elements of educational success based, above all, on the qualities of a competent teacher capable of adapting his intervention concerning the characteristics of the educational system in which it is inserted (35).

Starting from the previous theoretical models, the central idea emerges that the subjects of educational success can be considered as non-linear systems so that a slight modification of one of the systems (micro, meso, exo, macro) that interact with each other can cause significant changes in their way of perceiving and interpreting physical-sports education generating different approaches and solutions that the teacher must be able to mediate through his pedagogical, didactic-methodological strategies (36). *Teaching swimming for understanding (TSfU)* and multiteaching styles represent key elements in transferring previous concepts into a more contemporary and current interpretation of teaching swimming to children. *Teaching swimming for understanding* interprets aquatic exercise as a game through metaphors and variations that facilitate the mastery and understanding of the principles and strategies necessary for functionally utilizing the aquatic environment, thereby enhancing perception and control of one's body and the mechanical and anatomical laws that favour govern aquatic motor control.

Simplification of practice, self-organizing skills, and spontaneous learning stimulated through a dialogic approach, generating contrasting and varied situations facilitating learning, connecting concrete experience with abstract concepts through a reflective approach, encouraging work in small groups and dialogues that promote relationships and sharing of discoveries, and building generalizable movement structures represent the guiding criteria of this methodology (32, 37). This way, multiteaching styles represent a synthesis of all the previous elements based on how to teach (38). It is characterized by an integration related to the use of different teaching styles and the instructor's ability to manage groups in a broader context that includes awareness of the environment and all the sociocultural factors and elements that influence it. To the best of our knowledge, few studies nowadays have investigated in a holistic vision the effects of the teaching styles, and instructors' personal and didactical competence on children's competence and self-perception in swimming. Moreover, no studies have been conducted considering and analysing the integration of contemporary learning during swimming classes (4).

This study highlights the limits of assessing swimming instructors’ (SI) effectiveness solely through their didactic, methodological, and personal competences (39). Learning outcomes cannot be predicted exclusively from instructors' skills or from children's perceived and actual competences, as these are also shaped by the relational dynamics established during the teaching process. External observation by trained researchers provides reliable and valid data, offering a more accurate picture of the teaching process through key indicators such as active vs. inactive time, and moments of reflection and interaction. These quantitative and qualitative measures allow a deeper understanding of both technical (learning to swim) and formative achievements (40). Without such observation, instructors risk relying on rigid strategies that reduce adaptability to learners' needs. Systematic data collection across different contexts enables the construction of an increasingly objective framework of pedagogical practices, supporting the transfer of effective methods into specific teaching situations. The present research is situated within this descriptive–observational perspective.

**Figure 1:**
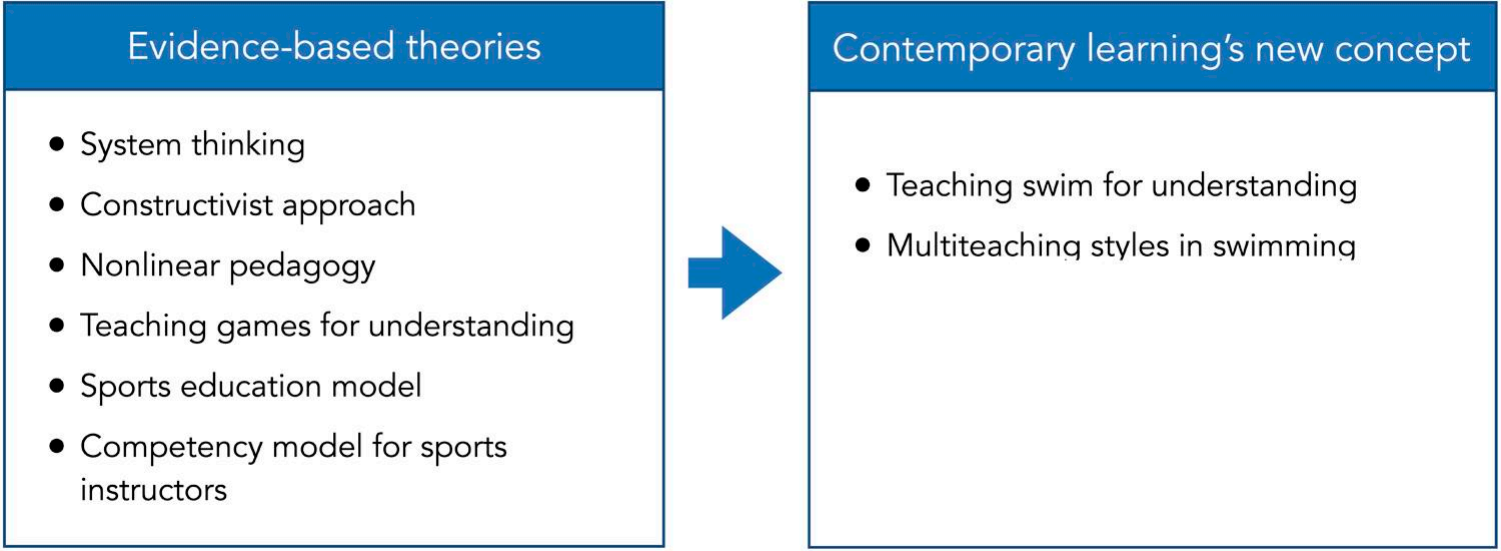
Framework of the new concept of contemporary swimming learning.

**Figure 2:**
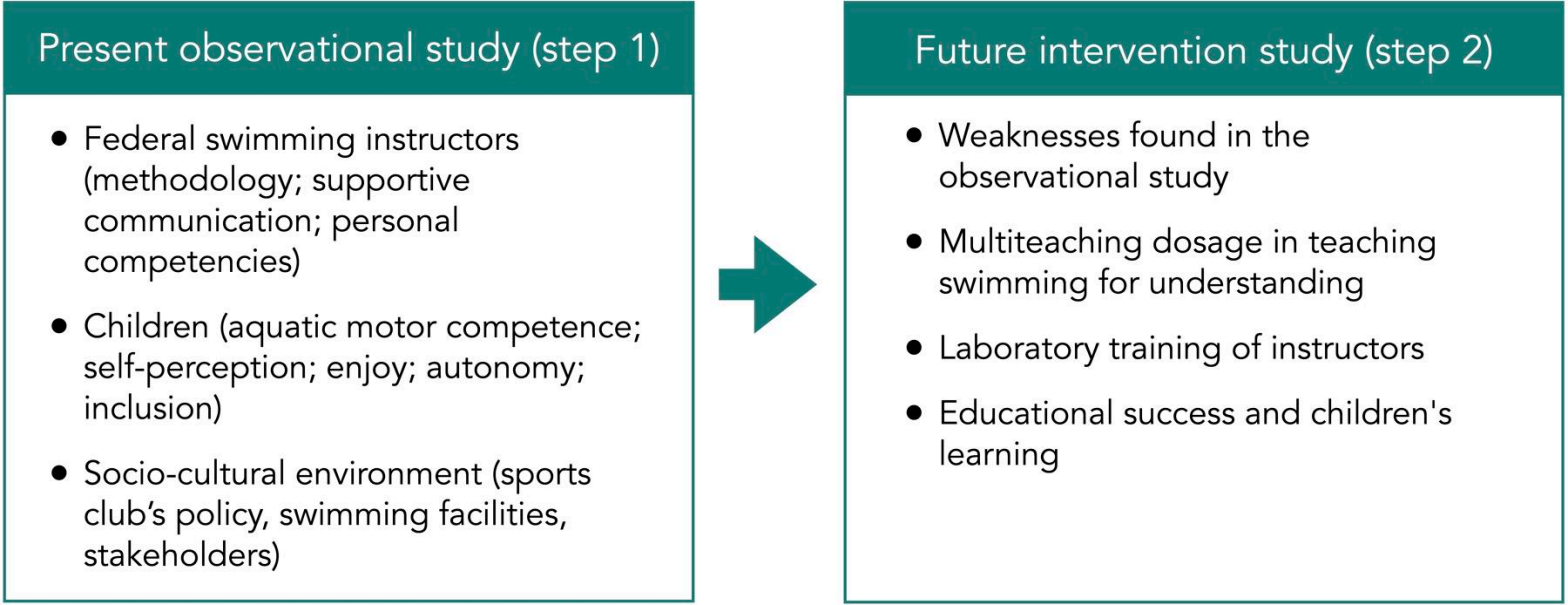
Research roadmap.

Considering the aforementioned aspects (Figure 1), the direction of our research, after an initial preliminary analysis aimed at collecting data on the methodological approaches of the swimming instructors and the effects of their teaching (Figure 2, Step 1 = the present study), will be extended to other elements of the systems thinking (Figure 2, Step 2 = future study). They include the training of instructors provided by a university's staff aimed at the overall development of the child and their skills (41, 42), also in a cognitive and psychosocial contexts based on *teaching swimming for understanding* and to the appropriate use of multiteaching styles considering the specific characteristics of the environment.

The present study aimed to analyse the personal (empathy and self-control), didactical, and methodological competencies of swimming instructors, investigating their relationships with aquatic competence and self-perception of children enrolled in swimming courses, as they characterize the “unity of analysis”, comprehensive of main elements favouring aquatic learning. It has been hypothesized that swimming instructors at present prevalently use a traditional teaching swimming approach based on linear and monoteaching approaches, as evidenced by the literature (4).

## 2 Materials and methods

The participants in the study are the swimming instructors of a large sports club in the hinterland of Milan and the children enrolled in the swimming courses they conduct.

Two hundred children attending swimming lessons, aged between 5 and 8 years (females, 94; males, 106; age, 6.0 ± 1.7 years; weight, 20.8 ± 4.6 kg; height, 1.2 ± 0.1 m; body mass index, 15.2 ± 1.7 kg/m^2^) were recruited to participate in the study. The sample size was calculated using the G*Power program (version 3.1.9.4), with a statistical power of 95% and an estimated effect size of rho = 0.3. The correlation was chosen for this calculation (*t*-test, correlation: point biserial model, one tail, alpha = 0.05). To achieve the previously set statistical power, 111 participants were required. However, 200 participants are recruited to consider any “dropouts”. The inclusion criteria were participation in the introductory swimming course and possession of the necessary medical certificate attesting fitness for sports. The exclusion criterion was the lack of consent to participate in the study and to data processing.

The study also involved 44 swimming instructors who hold the federal patent (21 females, 23 males; age 31.7 ± 8.3 years, teaching experience 4.7 ± 3.5 years) who volunteered to participate in the study. The statistical power from this sample size resulted in 95%. As the principal measures of swimming instructors were analysed using the two-way ANOVA, the statistical power was evaluated considering the *F* test “ANOVA: repeated measures, within factors”, using an effect size *f* = 0.25, an alpha value of 0.05, one group, and three repeated measures (as it would be the lowest number of repeated measures considered in the analysis).

The learn-to-swim school where the study was conducted has as its main goals to teach swimming skills for water safety and drowning prevention using a traditional approach. More specifically, the courses led or attended by participants aim at mastering prerequisites for water confidence and basic aquatic locomotor skills. In the specific context of the present study, instructors taught at the edge of the pool.

The swimming centre is affiliated with the FIN (Italian swimming federation) swimming school. There are three pools (beginners, recreational, and 25 m). The beginner's pool is 0.7 m high, 6 m long, and 4 m wide, with a water temperature held between 29°C and 30°C. The beginner's pool is split in the middle by a floating cable. The recreational pool is 1.1 m high, 12 m long, and 6 m wide, longitudinally divided into three lanes by floating cables. The recreational pool's water temperature is held between 28°C and 29°C. The third 25 m pool (12 m wide, divided into six lanes, depth 1.2–1.8 m) was not used by the children of the present study. The pool air temperature is 27 °C. The centre offers 45 min courses, once a week, for 36 weeks per year. The courses follow one another continuously; hence, to avoid wasting time, dedicated staff members (children's attendants) assist children in changing clothes. Furthermore, attendants guide children from parents to instructors and vice versa. Parents can observe the course lessons from the stands within the pool facility. Teachers have available traditional swimming tools such as swimming boards, pull buoys, floating tubes, armrests, floating carpets, and sinkable objects.

This study was approved by the ethical committee of the University of Milan on 12 March 2025 with opinion number 39/25.

### 2.1 Measures

#### 2.1.1 Instructors: teaching styles, self-control, empathy, didactic competences

Measurements related to the instructors' teaching approach were accomplished through:

- The Teaching Styles Questionnaire (TSQ) (43, 44) using the teaching style spectrum method of (45). It is an instrument designed to examine instructors' beliefs about teaching style methods used in their sports teaching. The questionnaire presents a five-point Likert scale score for each teaching style. Points near 5 indicate frequent use of the teaching styles, while points near 1 mean they are not used. Scores higher than 3 represent frequent use of the teaching style, as indicated by the descriptors, while scores of 3 or lower represent sporadic or no use of them. To indicate a prevalent use of a teaching style, values higher than or equal to four were considered. Moreover, as literature indicates that in traditional swimming teaching, instructors frequently use up to three teaching styles (5, 6), values higher than or equal to four were considered as multiteaching for this specific study.
- The Self-Control Questionnaire (SCQ) (46). It describes the self-estimated instructors' level of self-control. The questionnaire consists of 36 items, each rated on a five-point Likert scale, ranging from 1 (“not at all like me”) to 5 (“very much like me”). As in literature, no qualitative cut-off has been identified for this scale; scores higher than 60% of the total score were considered fair levels of self-control (as it would be higher than the total score resulting from the sum of the neutral items of the Likert scale).
- The Empathy Scale for Teachers (EST) (47). It is a self-evaluation questionnaire designed to measure the level of empathy among instructors. The questionnaire consists of 19 questions; each is rated on a four-point Likert scale. As in literature, no qualitative cut-off has been identified for this scale; scores equal to or higher than 60% of the total score were considered fair levels of empathy (as it would be higher than the total score resulting from the sum of the neutral items of the Likert scale).
- The Internship Evaluation Sheet in Physical Education and Sports (IESPES) (42, 48, 49). It is an instrument designed to measure the different teaching knowledge acquired through real practice in the field highlighted in the application context of the swimming lessons. The main areas evaluated were the instructor's communication, didactic organization, and motivation capacity. Each item was evaluated using a descriptor schedule and could obtain a score from 0 to 5. Scores higher than three are considered a fair level of didactical competence, as competence scores 4 and 5 are higher than the neutral descriptor (3 points).
- The Instrument for Identifying Teaching Styles (IFITS) (50). This tool was developed to identify the prevalent teaching styles used, analysing video-recorded lessons. During the video analysis, the rater indicates the teaching style used by the instructor every 20 s. At the end of the video analysis, the instrument reported the percentage of use for each teaching style during the lesson.
- The System for Observing Fitness Instruction Time (SOFIT) (51). This tool aims to evaluate the amount and type of physical activity performed during the lesson. Information about the lesson content and the quality of the instructor's interaction with children is also provided. A rater evaluates the lesson recorded with a dedicated schedule, giving every 20 s information about the type of physical activity (laying, sitting, standing, moderate activity, vigorous activity), the content of the lesson (management, knowledge, game, skill, fitness, or other), and the interaction of the instructors to promote physical education practice (interaction with inside lesson topic, interaction with outside lesson topic, no interaction). At the end of the evaluation, the percentages of the type of physical activity, lesson content, and the instructor's interaction are provided for each variable analysed.

#### 2.1.2 Children: actual and perceived aquatic competence, psychosocial perception

The actual and the perceived aquatic competence and enjoyment, inclusion, autonomy, and self-efficacy of the children participating in the study were respectively measured through:

- The Pictorial Scale of Perceived Water Competence (PSPWC) (52). The tool administered by the researchers to the instructor allows for assessing the actual children's aquatic abilities. The instructor must identify the image that best corresponds to the specific aquatic ability among the given options. Each increasing degree illustrated by the images is associated with a score ranging from 1 (not able) to 3 (able). The mean of the values represents the final score, ranging from 1 (low level of water competence) to 3 (high level of water competence).
- The Aquatic Perceived Competence Pictorial Scale (APCPS) (53). It is a perception scale that examines the child's perception of swimming in terms of autonomy (e.g. “At the swimming pool, how do you wear your swimsuit, cap, and bathing shoes?”), enjoyment (e.g. “How do you feel when you go to the swimming pool?”), inclusion (e.g. “Do your friends choose you to play in the water?”), self-efficacy (e.g. “What position do you think you would finish in a water race with your friends?”), and perceived aquatic competence. These are fundamental themes for educational and sports success, and they are grounded in the principles of self-efficacy and self-determination (53, 54). Each increasing degree, illustrated by the images, corresponds to a score of 1–3. The final score is represented by a value ranging from 1 (low perception of aquatic ability) to 3 (high perception of aquatic ability). The researchers administered the APCPS to the children before a swimming lesson halfway through the swimming lessons: the children completed the written form of the scale by marking the figure that, for each item, best reflected their experience.

As the APCPS also considers children's autonomy, enjoyment, inclusion, and self-efficacy in addition to the perceived aquatic competence, the scale was preferred to be administered to the children. The PSPWC was preferred to be administered to the instructors to assess the children's actual aquatic competence.

The PSPWC by the teachers and the APCPS by the children were collected on the same day.

### 2.2 Procedures

The tests were administered during the practice hours of swimming courses and were conducted by the university research team. The questionnaires have been administered halfway through the swimming course. In the same period, three lessons for each child group were video recorded, and the IESPES, IFITS, and SOFIT were analysed. The inter- and intra-rater reliability was assessed to ensure the validity of the measures. To evaluate the intra- and inter-rater reliability of IESPES, SOFIT, and IFITS, three video recordings of three instructors' observations and three video recordings of three children's lessons were used. Three different researchers who were adequately trained analysed the videos. Researchers repeated the evaluation every 2 weeks. The video sequence was randomly assigned.

### 2.3 Statistical analysis

SOFIT and IFITS reliability was assessed using the percentage of agreement by applying the following formula: % agreement = n° agreement/(n° agreement + n° disagreement) × 100. An agreement rate of over 80% was deemed necessary for the test to be considered reliable. IESPES reliability was assessed using the interclass correlation coefficient (ICC).

All data resulted in a non-normal distribution. The item comparison of the results of questionnaires, tests, and video analysis for both instructors and children was performed using the non-parametric Friedman test. The *post hoc* was performed using the Bonferroni correction. The effect size (Kendall's *W*) was assessed (effect: <0.1 = very small; 0.1–0.3 = small; 0.3–0.5 = moderate; ≥0.5 = large). Differences between perceived aquatic competence and actual aquatic competence were assessed with the Wilcoxon test, and the effect size was calculated using the Wilcoxon effect size (*r*) with the following cut-off: small (0.10–0.3), moderate (0.30 to <0.5), and large (≥0.5). Moreover, the main results of the instructors’ questionnaire were correlated by analysing Spearman's rho. The same analysis was performed by correlating the children's aquatic competence and their perception of competence with the results of their instructors' questionnaires. The alpha value was set at 0.05.

## 3 Results

### 3.1 Reliability

The IESPES obtained values of intra-rater ICC of 0.993 and inter-rater ICC of 0.982. IFITS and SOFIT evaluations obtained values of agreement of 81% for intra-rater reliability and of 87% for inter-rater reliability.

### 3.2 Descriptive statistics

#### 3.2.1 Swimming instructors

**Figure 3:**
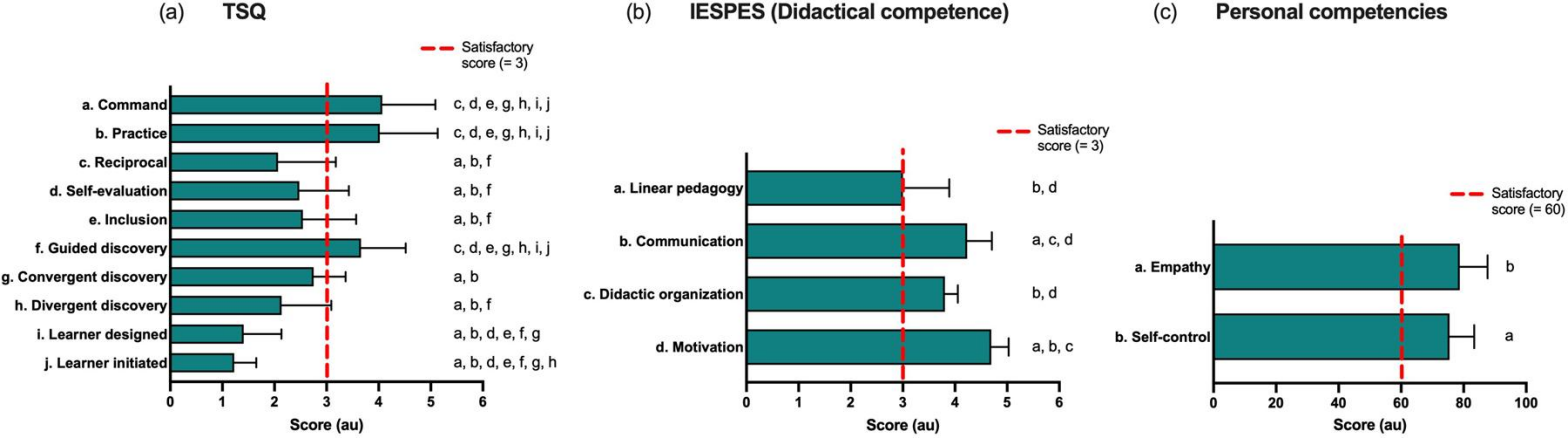
Instructors’ methodological, didactical, and personal competencies. **(a)** Teaching Style Questionnaire (TSQ); **(b)** Internship Evaluation Sheet in Physical Education and Sports (IESPES); **(c)** personal competencies (empathy and self-control). The red dotted lines display the satisfactory score levels. The letters indicate what item is significantly different (*p* < 0.05) in the paired comparison (letter = different than).

In TSQ, instructors reported a prevalent use of the command (4.1 ± 1.0 au, *W* = 0.61), practice (4.0 ± 1.1 au, *W* = 0.61), and guided discovery (3.7 ± 0.9 au, *W* = 0.61) teaching styles (Figure 3a). The instructors' group reported a monoteaching tendency (2.9 ± 1.7 teaching styles prevalently used). The evaluation sheet (IESPES, Figure 3b) shows a good level of didactic competence as all values are over 2.5 au (considered the medium reachable value). Nevertheless, the didactic organization is the lower value (3.8 ± 0.2 au, *W* = 0.72). Moreover, the IESPES describes the instructors as a group with a prevalent linear approach (3.0 ± 0.9 au, *W* = 0.72). Finally, the evaluation of personal competencies showed more than sufficient levels of personal competencies, as self-control and empathy exceeded 60% of the maximum results reached by the instructors (Figure 3c).

The IFITS video analysis highlights that the command (41.1 ± 18.8%, *W* = 0.88) and practice styles (28.7 ± 15.9%, *W* = 0.88) are prevalently used; moreover, they reported a little use of the guided discovery (4.5 ± 7.1%, *W* = 0.88) and spent around a quarter of the time in management of the lesson (24.9 ± 8.1%, *W* = 0.88).

**Figure 4:**
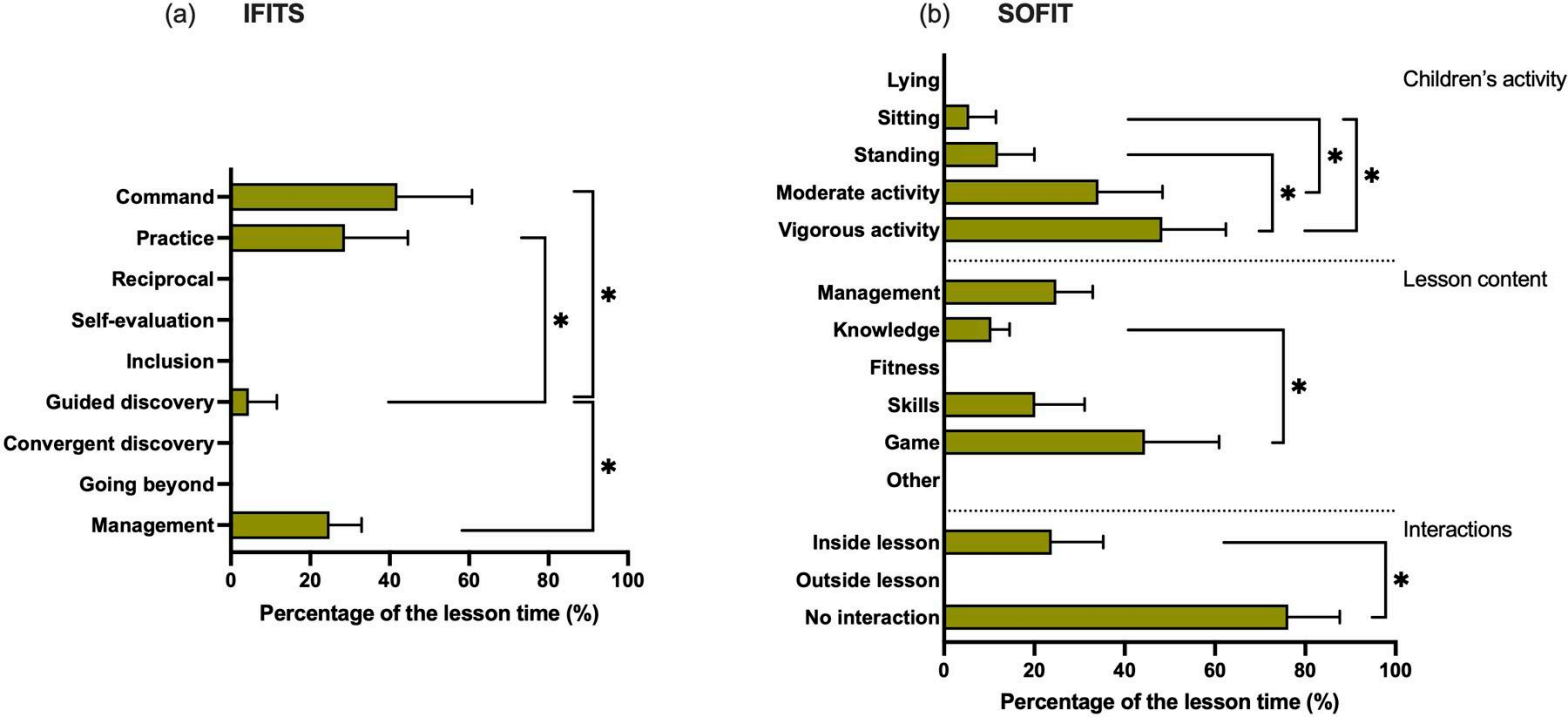
Instructors’ results regarding the methodological competencies from the instrument for identifying teaching styles (IFITS) and the system for observing fitness instruction time (SOFIT) analysis. **(a)** IFITS results; **(b)** SOFIT results. Significant difference: *p < 0.05.

SOFIT analysis highlighted that participants spent around half of the lesson in vigorous physical activity (48.3 ± 14.1%, *W* = 0.77), a third of the lesson in moderate physical activity (34.2 ± 14.2%, *W* = 0.77), and a fifth of the time inactive (overall inactive time 17.5 ± 11.3%, *W* = 0.77). The lesson contents are primarily oriented towards games (44.5 ± 16.4%, *W* = 0.87) and management (24.9 ± 8.1%, *W* = 0.87), while less time is spent on skill exercises (20.2 ± 10.9%, *W* = 0.87) and reflection moments (knowledge: 10.5 ± 4.0%, *W* = 0.87). Instructors poorly interact with children (no interaction: 76.2 ± 11.4%, *W* = 1.00). All the statistical comparisons and results are reported in Figure 4.

#### 3.2.2 Children

**Figure 5:**
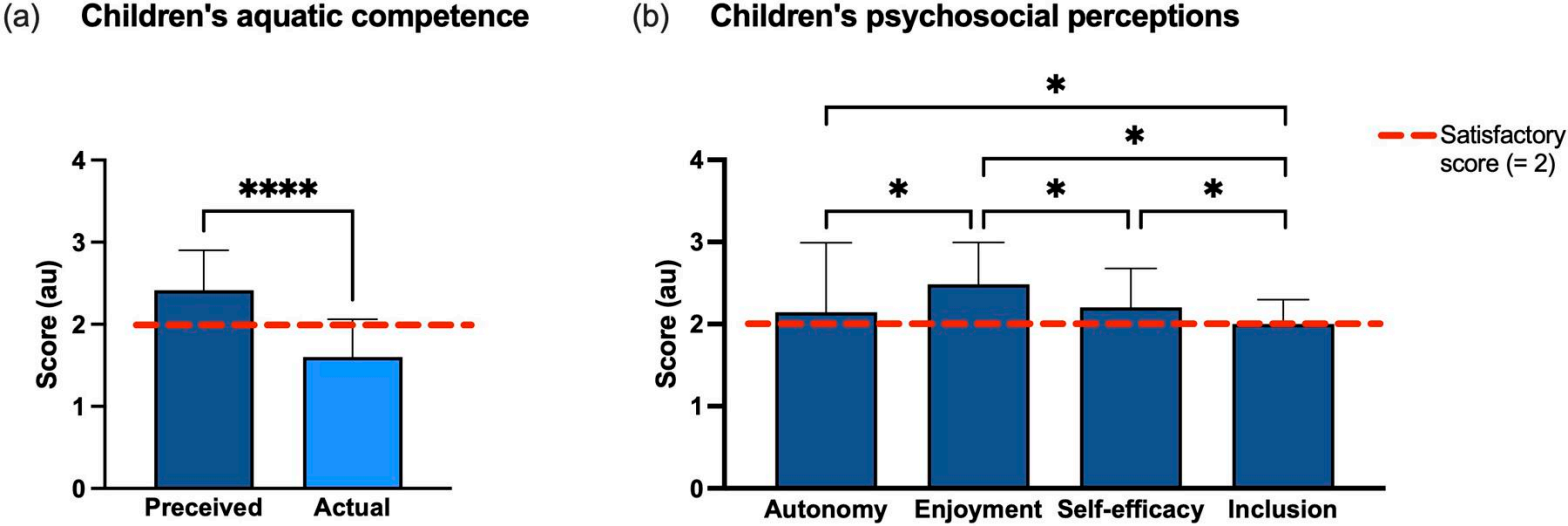
Results about children's aquatic competence **(a)** and children's psychosocial perceptions **(b)**. The red dotted line represents the satisfactory score considered (2 = satisfactory score). Significant difference: *p < 0.05; ****p < 0.0001.

Participants reported medium to high levels of perceived water competence (2.4 ± 0.5 au). Analysing the questionnaire's items, the group showed lower levels of perception of inclusion (2.0 ± 0.3 au) and autonomy (2.2 ± 0.8 au). The comparison between the PSPWC (actual aquatic competence) and APCPS (perceived aquatic competence) showed higher values in perception of competence (2.4 ± 0.5 vs. 1.6 ± 0.5 au, *r* = −0.83). The results are reported in Figure 5.

### 3.3 Correlations

**Figure 6:**
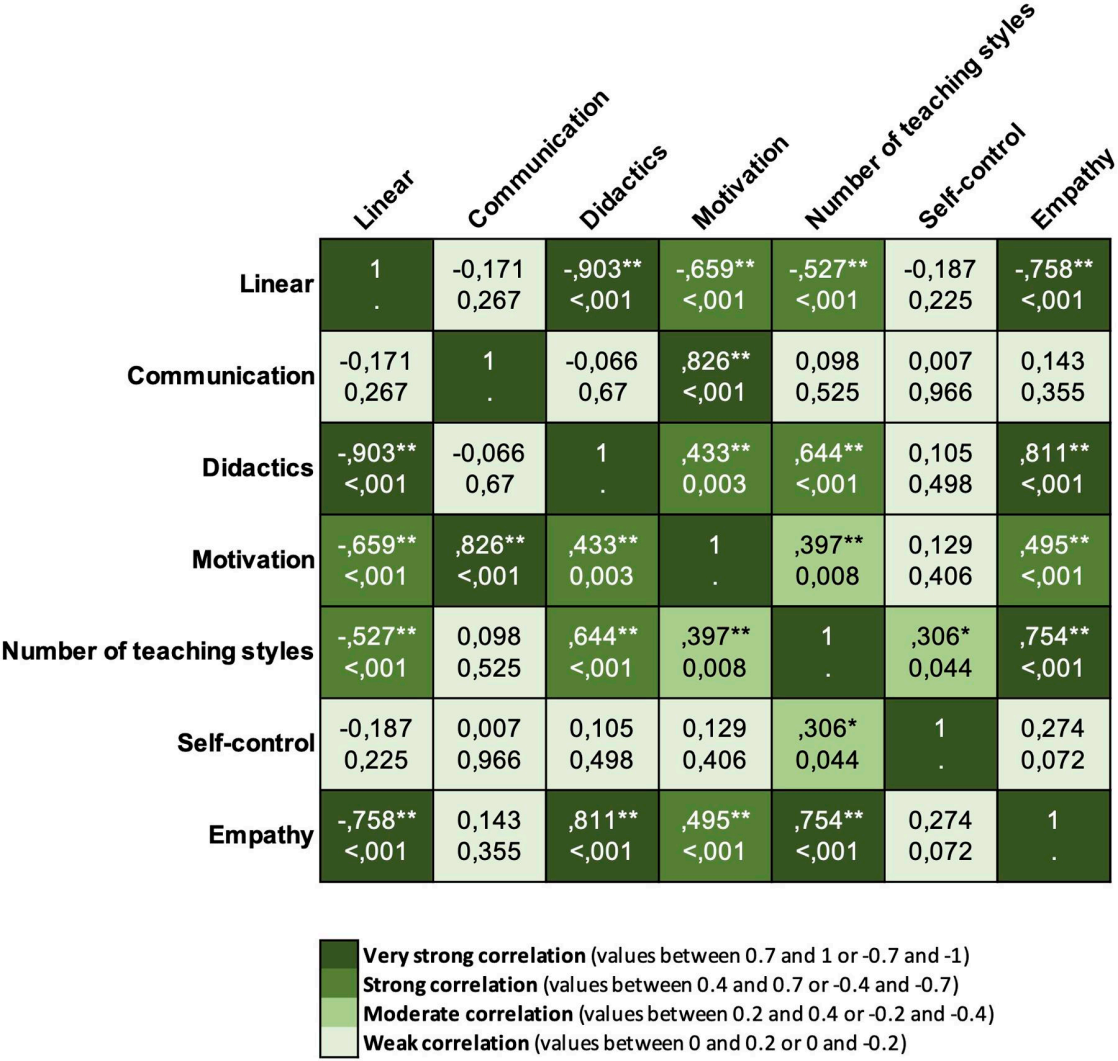
Heatmap of the correlations between variables related to the instructors’ teaching approach.

The linear pedagogy level showed an inverse and strong correlation with IESPES (didactical competencies) variables (except for the variable communication, which was not correlated), the number of teaching styles used, and the level of instructors' empathy. Conversely, the number of teaching styles used (methodological competencies) is directly strongly correlated with IESPES didactic organization and moderately with motivation values (didactical competencies). Moreover, the number of teaching styles used is also moderately correlated with the level of instructors' self-control and strongly with empathy (personal competencies). Correlations' strength and significance are reported in the heatmap (Figure 6).

The PSPWC (actual aquatic competence) resulted directly and strongly correlated with APCPS (perceived aquatic competence). Moreover, children's PSPWC (actual aquatic competence) is also moderately directly correlated with the values of their instructors' IESPES didactic organization and motivation and inversely correlated with their level of linear pedagogy. In addition, the children's PSPWC (actual aquatic competence) is directly correlated with their instructor's number of teaching styles (strong) used and empathy (moderate).

**Figure 7:**
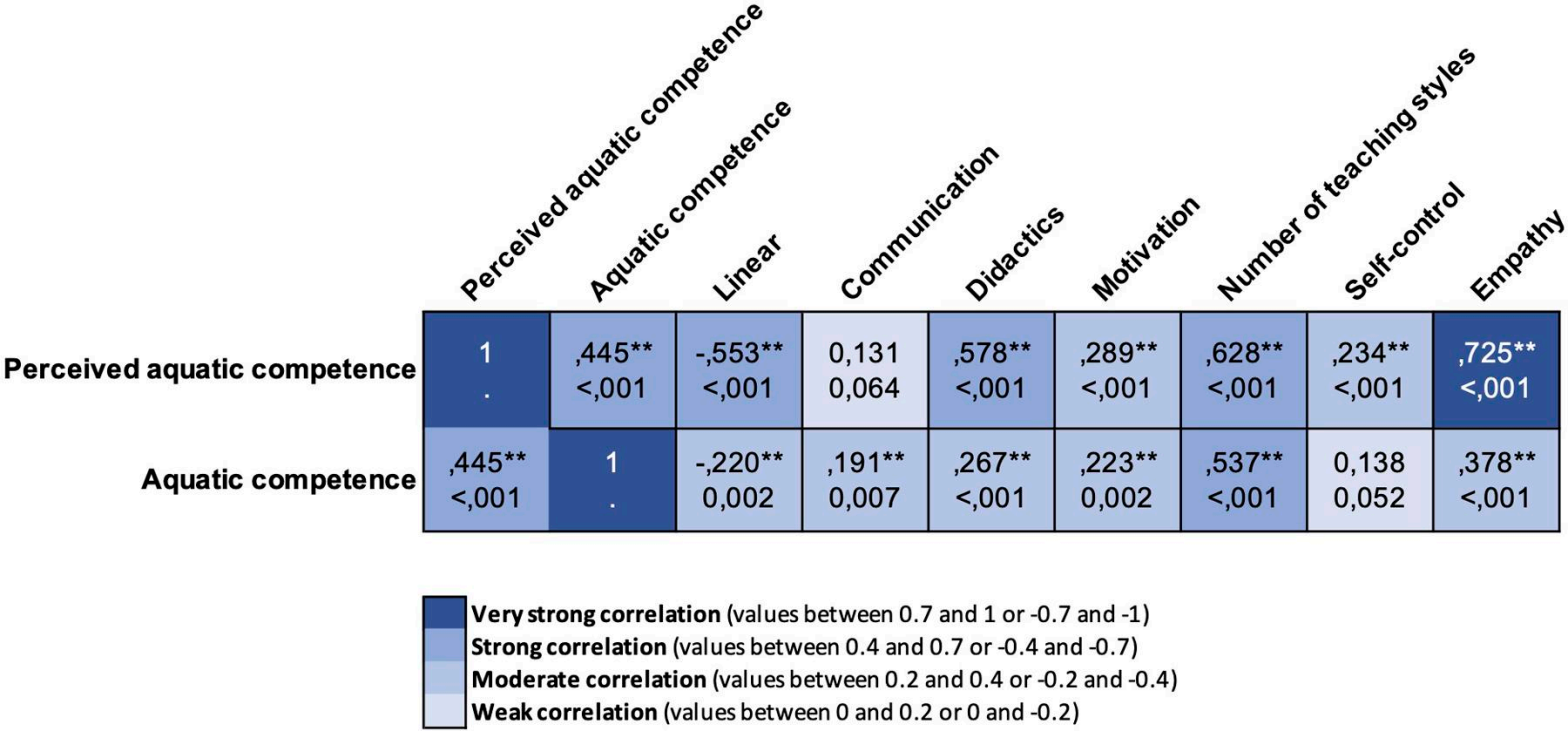
Heatmap of the correlations between children's perceived and aquatic competence and variables related to the instructors’ teaching approach.

Considering the children's APCPS (perceived aquatic competence) resulted in an inverse correlation with their instructors' level of linear pedagogy (strong) but directly correlated with variables of IESPES (didactic organization = strong; motivation = moderate). Moreover, children's APCPS (perceived aquatic competence) is directly correlated with the number of teaching styles used by their instructor (strong), self-control (moderate), and the level of empathy (strong). Correlations' strength and significance are reported in Figure 7.

## 4 Discussion

The main purpose of this study was to verify whether the didactic-methodological skills of swimming instructors are effective for children's educational success, oriented towards contemporary learning based on current scientific evidence, and follow the law indications (Article 33 of the Italian Constitution), which place the child as an active subject of his learning. Additional aims were (a) verification of the swimming instructors' relationship between the different skills (didactic, methodological, personal) and (b) verification of the relationships between the number/type of teaching styles used by instructors, the children's aquatic skills, and their perception of their own skills and of the swimming pool environment in which they carry out the activity.

The results from the TSQ demonstrate that the instructors have a good awareness of the type of methodology they used. In fact, these results are confirmed and integrated by the video analyses (IESPES, IFITS, and SOFIT), highlighting similarities to TSQ answers, reporting the use of prevalent monoteaching pedagogy and mostly linear teaching styles focused on command and practice styles. Furthermore, their personal competences (empathy and self-control) were more than adequate, as well as their didactical organizational skills. Children exhibited a discrepancy between actual and perceived aquatic competence, the latter overestimating the former.

The observation of the lessons, based on inter- and intra-rater reliability, showed excessive time spent by instructors in managing collective actions (e.g. departures one at a time while the other children are still; attention focused on some children while the others are inactive), which implies dead and inactive times (24.9% of the lesson). Compared to previous studies by the present research group, which analysed teachers' conduct while teaching in different contexts but under a similar scientific investigation approach (38, 42, 55, 56), the SI appear to spend more inactive time than physical education specialist teachers (PES) and instructors of other sports (RI) (ranging from 3.9% to 8.5% and from 3.0% to 7.8%, respectively; Table 1). Conversely, swimming instructors' inactive time (SI), as reported in the previous studies, was approximately 2.8%–3.4% lower compared with the generalist (G) teachers. Although these data represent an important indication of the need to reduce downtime, they must nevertheless be normalized in the aquatic context. It is interesting to note, however, how a multiteaching and multi-activity approach generally significantly reduces this type of timing (38, 42, 57, 58). Specifically, in rugby instructors, inactive times reduce from 7.5% to 3.0% (56).

**Table 1:** Dose–response extrapolated by previous studies on traditional and contemporary learning.

Reference	Context	Instructors and teachers	Instructors’ and teachers’ teaching approach	Lesson composition				Motivation to practice			Pedagogical approach		
				In-action time	Inactive time	Reflection time	Other	Interaction outside the lesson	Interaction inside the lesson	Other (no interaction)	Linear	Non-linear	Other
The present study	Learn-to-swim children	SI	Common sport practice (Monoteaching)	48.3	24.9	10.5	16.3	0.0	23.8	76.2	70.6	4.5	24.9
Invernizzi et al. (38)	Primary school	PES	Multiteaching	78.9	3.9	17.2	0.0	N/A	N/A	N/A	N/A	N/A	N/A
		G	Monoteaching	72.3	27.7	0.0	0.0	N/A	N/A	N/A	N/A	N/A	N/A
Invernizzi et al. (55)	Primary school	PES	Multiteaching NL	74.3	8.5	17.2	0.0	N/A	N/A	N/A	20.5	79.5	0.0
		PES	Multiteaching LI	83.7	4.3	12.0	0.0	N/A	N/A	N/A	82.2	17.8	0.0
		G	Monoteaching	71.7	28.3	0.0	0.0	N/A	N/A	N/A	23.8^a^	0.0	76.2^b^
Rigon et al. (42)	Rugby children	RI	Common sports practice (monoteaching)	66.9	7.8	21.6	3.7	0.0	38.5	61.5	60.9	0.0	39.1
Rigon et al. (56)	Rugby children	RI + E	Multiteaching	74.4	3.0	23.0	0.0	4.4	42.1	53.5	70.9	10.5	21.4
		RI	Monoteaching	74.6	7.5	17.9	0.0	0.0	38.0	62.0	68.3	0.0	31.7

All values are expressed in percentages (%) of the lesson. SI, swimming instructor; PES, physical education specialist; G, generalist; RI, rugby instructor; RI + E, rugby instructor receiving additional specific education.

^a^ Prescriptive style.

^b^ Free play (without pedagogical goal).

This way, it is important to consider an appropriate choice and dosage of teaching and methodology in relation to the educational needs and problems. Dead and inactive times represent a critical point in sport education: from a didactical point of view, they lead to an inappropriate management of the lesson and a subsequent demotivation for children. Moreover, they reduce lessons' active time, limiting the specific experiences and reducing children's learning opportunities (59). In contemporary learning, the fair integration of in-action, reflection, and interaction time within the didactic moment is of great relevance, considering the high variability of the situated contexts to which this refers (60). In the present study, SOFIT analyses highlighted that <50% (48.3%) of the time of the lesson corresponded to in-action time (vigorous and moderate). These data highlight that the percentage of active practice is lower, from 18.6% to 35.4% than the results by Invernizzi et al. (38, 55) and Rigon et al. (42, 56), as reported in Table 1.

Referring to the scarcity of useful stimuli for health that emerge in this study (high inactive and low in-action times), several current studies (61) consider the importance of a multicomponent structure that takes into account the relationship with different stakeholders and institutions to promote an adequate amount of daily and weekly physical activity and the level of physical valuable activity for health (62).

Concerning reflection time, only approximately 10% (10.5%) of the proposals highlight reflective and dialogical learning, which promotes better perception and self-awareness in the aquatic environment. Hence, swim instructors present values that are lower from 1.5% to 12.5% compared with multiteaching prepared teachers and instructors (Table 1). In cognitive (63) and social constructivism (64), the construct of knowledge and the social factor is based on “disequilibrium” through contrasting and varied situations (differential learning) that connect concrete experience with abstract concepts through a reflective approach (questions) and the encouragement of work in small groups and dialogues that promote relationships and sharing of discoveries (65–68). Spending more time on reflection using more non-linear pedagogy and production styles leads to greater cognitive engagement and intervention of executive functions, conditioned by the amount of information that must be selected, perceived, and processed, as well as by the expansion of choices regarding possible responses and probable adaptations and adjustments (69, 70). This approach can imply a transfer based on processing theory (71) and a similarity of cognitive process elements that help stimulate not only physical motor skills but also psychosocial and cognitive ones.

Referring to the motivation to practice, SI outside lessons interaction aimed at promoting aquatic practice in other environments or aimed at promoting other physical and sporting activities beneficial for health is absent (0%), indicating a poor idea by the instructors of the concept of transference as a key element of long-term learning and physical literacy (72), which is together with retention an accurate and significant measure of learning (73). Team sport instructors who use a multisport and multiteaching (RI + E) methodology employ this type of interaction 4.4% of the time (56) (Table 1). The SI inside lesson interaction aimed at motivating the activity (23.8%) is 14.7% less than that of rugby instructors in common sport practice (42), as shown in Table 1. Considering that the results of IESPES indicate that SI have a high ability to motivate (Figure 3c), it is hypothesized that the stable and predictable environmental aspects characterizing the pool influence the results related to their interaction possibilities. Hence, a constructivist approach aimed at changing the physical pool environment could affect the dyad learner–instructor (2). The adoption of a predominantly linear monoteaching style, combined with the structural configuration of the pool in which the lessons took place (recreational, with lanes), may account for the predominance of communication oriented primarily towards the mere execution of exercises rather than fostering a reflective and interactive approach with the learners (2, 74).

In summary, the paucity of reflection and interaction time, combined with high inactivity and low action times, results in a poor stimulus to create adequate opportunities for learning and formative success.

The instructors' personal competencies represent a variable that contributes to adapting the teaching methodology to different social environments, physical activity contexts, and policies (32, 75). Indeed, current ecological dynamics and neuroscience scientific paradigms enhance a dynamic and interactive orientation of teaching processes by integrating learning approaches and instructors' pedagogical sensitivity on the specific sociocultural context (32, 33, 75–77).

The questionnaires analysing the SI’s empathy and self-control highlighted their positive personal skills (excellent and more than sufficient). Empathy was directly correlated with children's perceived aquatic competence (including perceptions of the environmental context) and their actual aquatic competence. Noticeably, compared with the latter, which has low to medium values, the children's perceived competence is overall medium to high. The difference between actual and perceived motor competence is an indicator of limited cognitive capacity development necessary to objectively evaluate one's capacities (78). Consequently, interventions aimed at increasing conscious actual motor competence (with reflective practice) can contribute to balancing children's self-perception (78). At the age range of the children observed in this study (5–8 years), reflective practice can be promoted through appropriate learning strategies. Specifically, children between the ages of 5 and 7 can think in terms of classes and relationships and carry out processes of generalization. Their thinking is characterized by magical thinking, artificialism, and finalism. For this reason, the use of imaginative storytelling becomes the preferred way to stimulate reflective functions through a dialogical and narrative approach, fostering a “guided discovery” of the self within the aquatic environment. From the age of 6–7—when children begin to access formal schooling—their ways of using thought become more complex. They gradually develop the ability to think in a decentred and reversible way; around the age of 7, they also acquire the assimilation of spatial and temporal categories, which guide them more effectively towards solving motor problems through both convergent and divergent discovery processes, related to the possibilities of using the “aquatic body” (63, 79).

Other specific studies (32) have highlighted how the subjective and dynamic relationship between instructors and learners is a determining factor influencing coordination patterns regardless of the kind of activity practice (80). It supports the teacher's empathy and personal skills as key elements to stimulate children's joy and intrinsic motivation, favouring the acquisition of aquatic competence through dialogue, questioning, and active reflection (81). This constructivist approach fosters connections and transversal links that can also be transferred to other learning areas, including the cognitive component (2, 10, 70). A supportive communication favouring these relational processes (82, 83) might constitute a useful way to improve the medium-low level of the aquatic competence of the sample observed in this study.

Conversely, a predominantly prescriptive traditional approach can negatively impact learning and does not promote adequate psychosocial and psychomotor skills (84, 85). Our results support this assumption; indeed, the values of teachers' empathy were inversely correlated with the use of linear styles, and children's autonomy and inclusion, which emerged from the perception of competence, were among the lowest values observed. In this vision, as evidenced by the literature, the instructors' personal competencies become of great importance in their professional formation. Indeed, in the present study's sample, higher methodological competencies are related to higher instructors' didactical and personal competencies. However, it is interesting to note that a linear style is related to swimming instructors with lower methodological and didactical competence.

Further data confirming how a contemporary learning based on a multiteaching background denotes a key element for effective teaching and is represented by the positive relationships of the number of teaching styles used by SI with IESPES items (didactic organizational ability, motivation, and communication), which in turn correlate with the teachers' level of empathy. The use of an exclusively linear pedagogy, on the other hand, correlates inversely with all these factors. This way, the only swimming program that applied a non-linear pedagogy to 5- to 12-year-old children (4) demonstrated how this program is more engaging for children, while linear pedagogy was deemed more rewarding by parents. Hence, considering the complexity of the swimming teaching system, the multiteaching approach allows the creation of more bonds between the stakeholders included in the system (children, parents, instructors' methodology, etc.) (8, 16, 86).

The study by Invernizzi et al. (55) (Table 1) highlighted that increasing by approximately 18% non-linear pedagogy in the generalist's (G) traditional teaching (that was reported to be mainly by linear learning and recreational play) allows a significant advantage in children's psychomotor, cognitive, and psychosocial development. From this perspective, the dosages of teaching styles can yield different results depending on the specific context in which they are applied and the typology of the proposal activity (87, 88).

From a practical contextualization of the system ecological model by Bronfenbrenner and Coté (15–17) in addition to the micro- (children, instructors, swimming pools and tools) and meso-systems (behaviours emerging from their relationships and interactions) related to what is directly involved in the swimming teaching process, further elements that can negatively affect children's autonomy and inclusion are related to macro-systems (sociocultural aspects: building community culture, laws, organization policies, and instructor' training), and exo-systems (variables within swimming contexts not directly connected to the swimming teaching process: attendants, parents, facilities, sport centre vision/mission).

More specifically, if these elements reflect a managerial or exclusively connected to sport success mentality, they can generate wider social issues, such as children's non-optimal educational learning approaches, including low autonomy, self-management, and inclusion. In particular, the strict and rigid organization of spatial environment (e.g. pools divided by longitudinal lanes that reduce the dialogue between peers and instructors), low variety of tools, low differentiated environmental situations that can facilitate the children's active discovery methodology, are some of the causes of the previously indicated psychosocial and psychomotor problems (2, 74, 89). Another issue is represented by the sports centre's organization, which, in many cases, considers the aquatic environment a “swimming factory” where swimming courses are immediately one after another. The attendants favour this organizational management, but they often impede the children's autonomy (which resulted in one of the lowest values in self-perception results) during the phase of clothes changing (90). The lack of stand barriers avoiding direct vision and verbal communication between children and parents can constitute a distracting factor and an obstacle for children's autonomy development (91). This issue might depend on a wrong behaviour of parents, both overprotective or showing incorrect expectations of parents, mainly oriented towards a limited traditional technical approach addressed to a specific learning of swimming, which does not, however, consider the learning stage most suited to the child's level and development period (37, 92, 93).

In contrast to parents' vision, the contemporary learning methods considering situated teaching (70), produce effects on psychomotor learning, on the involvement of mental, psychological and social factors, enhancing relationships with the environment, people, and a reflection on experience totally different compared with a linear, repetitive, structured and sequential approach, which is not easily transferable to other fields and experiential contexts (94, 95). Contemporary learning breaks the canons of traditional learning, as it takes into consideration the relationships that occur within the whole system. It is no longer an approach focused solely on motor skills and sports, where each part of the system is considered separately, but rather one in which each part plays a key role in fostering the relationships between the subsystems (micro, meso, exo, macro) (17, 86).

### 4.1 Limits

To the best of our knowledge, no previous studies have investigated the dose–response effect of the didactic methodologies in swimming for educational success as part of systems thinking. Therefore, the present study has been compared with previous research in different contexts, presenting literature that only partially represents the phenomenon. Moreover, the present research involved only one sports centre and cannot be considered fully representative and generalized without a careful analysis of the specific swimming realities. In addition, child participants belonged to a restricted age range that included only basic aquatic motor competence and not technical swimming skills. Expanding the age range and the level of participants would cover the whole spectrum of targets of swim-to-learn programs and provide more comprehensive information about children's learning and instructors' teaching competence.

## 5 Conclusion

Instructors of this observational cross-sectional study showed good personal and didactic abilities, primarily based on a traditional linear approach. In addition, a discrepancy existed between children's actual and perceived aquatic competence. The paucity of reflection and interaction time, together with high inactivity and low action times, reduces the stimuli to create adequate learning opportunities. Therefore, contemporary learning could improve instructors' professionalism and positively influence the children's perceived (autonomy and inclusion) and actual (skill acquisition) aquatic competencies.

In the swimming educational setting, which our study addresses, dose–response methodology is an entirely new concept, both from a qualitative and quantitative perspective. To effectively finalize the concept of dose–response from the standpoint of sustainability in the educational-methodological domain towards a formative success, it is crucial to consider the multiteaching style and the *teaching swim for understanding* as methodologies that better address learning affordances and any other environmental components (ecological approach) integrated with reflection and activity promotion inside and outside the lesson.

### 5.1 Future perspectives

**Figure 8:**
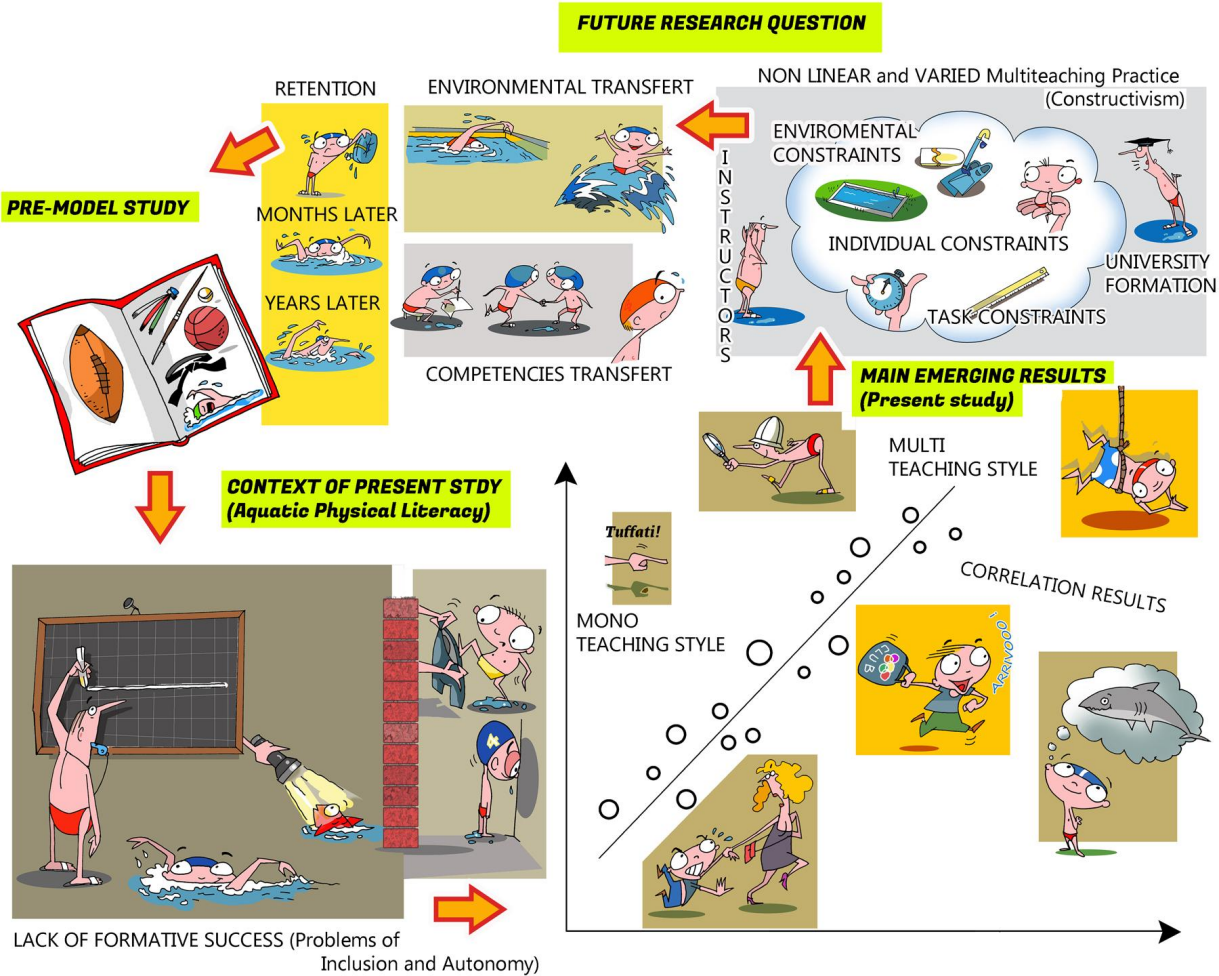
Resume of the main results of the present work (observational study) and future research perspective (intervention study).

The research project aims to analyze the effectiveness of an innovative university curricular model for the training of swimming instructors, an example of which is shown in Figure 8.

This model emphasizes the integration of methodological, didactic, and personal skills, regarded as interactive and interdependent elements, to evaluate their impact on the educational and sporting success of the children entrusted to the instructors. The research, which encompasses both descriptive–observational and intervention-based studies, is founded on collaboration among universities, instructors, and sports clubs to guide training towards a more pedagogically informed approach.

From this perspective, instructors are expected to adapt their teaching style in a “unit of analysis” to reduce inactive time, provide more effective feedback, diversify relational approaches, and strengthen supportive communication. This enables the creation of greater learning opportunities, enhances educational time, and fosters in children the development of physical literacy—valuable not only in sport but also in everyday life. The adopted approach, called “multiteaching”, is inspired by the model of the “teacher–researcher” and offers flexible, non-prescriptive guidelines, allowing freedom of choice among different teaching strategies and styles, adaptable to the needs and contexts in which instructors operate (41).

These concepts, combined with the findings of the observational study already conducted, provide a foundation for promoting new scientific awareness in the teaching of aquatic physical activities and for shaping future developments in swimming instructors' training.

## Data Availability

The datasets presented in this study can be found in online repositories. The names of the repository/repositories and accession number(s) can be found below: https://zenodo.org/records/15804612.

## References

[1] Kirk D Macdonald D. Situated learning in physical education. J Teach Phys Educ. (1998) 17(3):376–87. 10.1123/jtpe.17.3.376

[2] Light R Wallian N. A constructivist-informed approach to teaching swimming. Quest. (2008) 60(3):387–404. 10.1080/00336297.2008.10483588

[3] Risyanto A Subarjah H Ma'mun A Nuryadi Prabowo I. The effect of student-centred learning approaches in physical education on positive youth development. Edu Sport Indon J Phys Educ. (2024) 5(1):10–21. 10.25299/esijope.2024.vol5(1).14532

[4] Minkels C van der Kamp J de Vries R Beek PJ. Learning how to swim in 5- to 12-year-old children: a scoping review of evidence-based motor learning methods. Front Sports Act Living. (2025) 7:1505301. 10.3389/fspor.2025.1505301 40012856 PMC11861109

[5] Hanif AS Mardesia P. Teaching styles and motivation in learning breast stroke in swimming. Asian Soc Sci. (2014) 10(5):2–6. 10.5539/ass.v10n5p2

[6] Rusdi R Dlis F Lubis J Nata AD Whalsen W. The effect of teaching style practice, reciprocity, inclusion and learning motivation on butterfly swimming skills. Kinestetik J Ilm Pendidik Jasm. (2020) 4(2):63–9. 10.33369/jk.v4i2.12574

[7] Koorts H Salmon PM Swain CTV Cassar S Strickland D Salmon J. A systems thinking approach to understanding youth active recreation. Int J Behav Nutr Phys Act. (2022) 19(1):53. 10.1186/s12966-022-01292-2 35549726 PMC9097093

[8] Baena-Morales S Merma-Molina G Ferriz-Valero A. Integrating education for sustainable development in physical education: fostering critical and systemic thinking. Int J Sustain High Educ. (2023) 24(8):1915–31. 10.1108/IJSHE-10-2022-0343

[9] Fernando SYJN Marikar FMMT. Constructivist teaching/learning theory and participatory teaching methods. J Curric Teach. (2017) 6(1):110. 10.5430/jct.v6n1p110

[10] Chow JY Atencio M. Complex and nonlinear pedagogy and the implications for physical education. Sport Educ Soc. (2014) 19(8):1034–54. 10.1080/13573322.2012.728528

[11] Werner P Thorpe R Bunker D. Teaching games for understanding: evolution of a model. J Phys Educ Recreat Dance. (1996) 67(1):28–33. 10.1080/07303084.1996.10607176

[12] Barba-Martín RA Bores-García D Hortigüela-Alcalá D González-Calvo G. The application of the teaching games for understanding in physical education. Systematic review of the last six years. Int J Environ Res Public Health. (2020) 17(9):3330. 10.3390/ijerph17093330 32403272 PMC7246645

[13] Evangelio Caballero C Sierra-Díaz MJ González-Víllora S Fernández-Rio FJ. The sport education model in elementary and secondary education: a systematic review. Movimento. (2018) 24(3):931. 10.22456/1982-8918.81689

[14] Ben-xu Z Mei-zhuo F. Research on the components of competency for the instructor of leisure sports club. 2011 International Conference on Future Computer Science and Education (2011). p. 400–3

[15] Bronfenbrenner U. Ecological models of human development. In: Gauvian M, Cole M, editors. International Encyclopedia of Education. 2nd ed. Oxford: Elsevier (1994). p. 37–43.

[16] Coté J Strachan L Fraser-Thomas J. Participation, personal development, and performance through youth sport. In: Positive Youth Development Through Sport. Abingdon, OX: Routledge (2008). p. 34–45.

[17] Coté J Turnnidge J Murata A McGuire CS Martin LJ. Youth sport research: describing the integrated dynamic elements of the personal assets framework. Int J Sport Psychol. (2020) 51:562–78. 10.7352/IJSP.2020.51.562

[18] Monat J Gannon T. What is systems thinking? A review of selected literature plus recommendations. Int J Syst Sci. (2015) 4:11–26. 10.5923/j.ajss.20150401.02

[19] Toomey B Ecker B. Of neurons and knowings: constructivism, coherence psychology, and their neurodynamic substrates. J Constr Psychol. (2007) 20(3):201–45. 10.1080/10720530701347860

[20] Arnold RD Wade JP. A definition of systems thinking: a systems approach. Procedia Comput Sci. (2015) 44:669–78. 10.1016/j.procs.2015.03.050

[21] O'Connor J Alfrey L Payne P. Beyond games and sports: a socio-ecological approach to physical education. Sport Educ Soc. (2012) 17(3):365–80. 10.1080/13573322.2011.608940

[22] Morin E. From the concept of system to the paradigm of complexity. J Soc Evol Syst. (1992) 15(4):371–85. 10.1016/1061-7361(92)90024-8

[23] Correia V Carvalho J Araújo D Pereira E Davids K. Principles of nonlinear pedagogy in sport practice. Phys Educ Sport Pedagogy. (2019) 24(2):117–32. 10.1080/17408989.2018.1552673

[24] Moy B Renshaw I Davids K. The impact of nonlinear pedagogy on physical education teacher education students’ intrinsic motivation. Phys Educ Sport Pedagogy. (2016) 21(5):517–38. 10.1080/17408989.2015.1072506

[25] Morales-Belando MT Kirk D Arias-Estero JL. A systematic review of teaching games for understanding intervention studies from a practice-referenced perspective. Res Q Exerc Sport. (2022) 93(4):670–81. 10.1080/02701367.2021.1897066 34705604

[26] Tan CWK Chow JY Davids K. How does TGfU work?’ Examining the relationship between learning design in TGfU and a nonlinear pedagogy. Phys Educ Sport Pedagogy. (2012) 17(4):331–48. 10.1080/17408989.2011.582486

[27] Giardullo G Aliberti S Sannicandro I Fattore S Ceruso R. Karate game: using a playful and participatory approach to enhancing children’s social and motor perception during the developmental age. Phys Educ Theory Methodol. (2024) 24(4):539–44. 10.17309/tmfv.2024.4.04

[28] Hastie PA Wallhead TL. Operationalizing physical literacy through sport education. J Sport Health Sci. (2015) 4(2):132–8. 10.1016/j.jshs.2015.04.001

[29] Zhang J Xiao W Soh KG Yao G Anuar MABM Bai X The effect of the sport education model in physical education on student learning attitude: a systematic review. BMC Public Health. (2024) 24(1):949. 10.1186/s12889-024-18243-0 38566018 PMC10986141

[30] Jung H Choi E. The importance of indirect teaching behaviour and its educational effects in physical education. Phys Educ Sport Pedagogy. (2016) 21:121–36. 10.1080/17408989.2014.923990

[31] Chatoupis CC. Physical education teachers’ use of Mosston and Ashworth’s teaching styles: a literature review. Phys Educat. (2018) 75(5):880–900. 10.18666/TPE-2018-V75-I5-8292

[32] Lémonie Y Light R Sarremejane P. Teacher–student interaction, empathy and their influence on learning in swimming lessons. Sport Educ Soc. (2015) 21(8):1249–68. 10.1080/13573322.2015.1005068

[33] Meyers S Rowell KR Wells MR Smith BC. Teacher empathy: a model of empathy for teaching for student success. Coll Teach. (2019) 67:160–8. 10.1080/87567555.2019.1579699

[34] Gola G. Conoscere l’insegnamento attraverso il cervello. Prospettive di interazione tra neuroscienze e processi didattici dell’insegnante. Form Insegn. (2020) 18(2):64–74. 10.7346/-fei

[35] Taylor IM Ntoumanis N Smith B. The social context as a determinant of teacher motivational strategies in physical education. Psychol Sport Exerc. (2009) 10(2):235–43. 10.1016/j.psychsport.2008.09.002

[36] El Zaatari W Maalouf I. How the Bronfenbrenner bio-ecological system theory explains the development of students’ sense of belonging to school? SAGE Open. (2022) 12(4):21582440221134089. 10.1177/21582440221134089

[37] Invernizzi PL Rigon M Signorini G Alberti G Raiola G Bosio A. Aquatic physical literacy: the effectiveness of applied pedagogy on parents’ and children’s perceptions of aquatic motor competence. Int J Environ Res Public Health. (2021) 18(20):10847. 10.3390/ijerph182010847 34682596 PMC8535907

[38] Invernizzi P Crotti M Bosio A Cavaggioni L Alberti G Scurati R. Multi-teaching styles approach and active reflection: effectiveness in improving fitness level, motor competence, enjoyment, amount of physical activity, and effects on the perception of physical education lessons in primary school children. Sustainability. (2019) 11(2):405. 10.3390/su11020405

[39] Pan Y-H. Relationships among teachers’ self-efficacy and students’ motivation, atmosphere, and satisfaction in physical education. J Teach Phys Educ. (2014) 33(1):68–92. 10.1123/jtpe.2013-0069

[40] Keating XD Kulinna PH Silverman S. Measuring teaching behaviors, lesson context, and physical activity in school physical education programs: comparing the SOFIT and the C-SOFIT instruments. Meas Phys Educ Exerc Sci. (1999) 3(4):207–20. 10.1207/s15327841mpee0304_2

[41] Casey A Kirk D. Models-Based Practice in Physical Education. New York: Routledge (2020).

[42] Rigon M Signorini G Scurati R Trecroci A Colella D Formenti D Relationship between multi-teaching styles and didactics effectiveness on rugby instructors and minirugby players. Children. (2024) 11(11):1319. 10.3390/children11111319 39594894 PMC11592661

[43] SueSee B Edwards K Pill S Cuddihy T. Self-reported teaching styles of Australian senior physical education teachers. Curric Perspect. (2018) 38(1):41–54. 10.1007/s41297-018-0041-2

[44] Yap GLY. The effects of autonomy supportive intervention programme in PE lessons to promote continued participation in sports and physical activity. Educ Res. (2021).

[45] Mosston M Ashworth S. Teaching Physical Education. 1st ed. San Francisco, CA: Pearson Education (2008).

[46] Tangney JP Baumeister RF Boone AL. High self-control predicts good adjustment, less pathology, better grades, and interpersonal success. J Pers. (2004) 72(2):271–324. 10.1111/j.0022-3506.2004.00263.x 15016066

[47] Wang X Zhang L Peng Y Lu J Huang Y Chen W. Development and validation of the empathy scale for teachers (EST). Stud Educ Eval. (2022) 72:101112. 10.1016/j.stueduc.2021.101112

[48] Kirkpatrick DL Kirkpatrick JD. Evaluating Training Programs. San Francisco, CA: Berrett-Koehler Publishers (2006).

[49] Dewi LR Kartowagiran B. An evaluation of internship program by using Kirkpatrick evaluation model. Res Eval Educ. (2018) 4(2):155–63. 10.21831/reid.v4i2.22495

[50] Curtner-Smith MD. Instrument for identifying teaching styles (IFITS) [Online] (2010). Available online at: https://spectrumofteachingstyles.org/assets/files/articles/CurtnerSmith2001_IFITS.pdf (Accessed October 27, 2024).

[51] McKenzie T. SOFIT (system for observing fitness instruction time) description and procedures manual (generic version) [Online] (2015). (Accessed August 8, 2025).

[52] Morgado LS Martelaer K Sääkslahti A Howells K Barnett LM D'Hondt E Face and content validity of the pictorial scale of perceived water competence in young children. Children. (2022) 10(1):2. 10.3390/children10010002 36670553 PMC9856909

[53] Murcia JAM Pérez LMR. Aquatic perceived competence in children: development and preliminary validation of a pictorial scale. Int J Aquat Res Educ. (2008) 2(4):313–29. 10.25035/ijare.02.04.05

[54] Ryan R Deci E. Toward a social psychology of assimilation: self-determination theory in cognitive development and education. In: Sokol BW Grouzet FME Muller U, editors. Self-Regulation and Autonomy: Social and Developmental Dimensions of Human Conduct. Cambridge, UK: Cambridge University Press (2013). p. 191–207.

[55] Invernizzi PL Signorini G Rigon M Larion A Raiola G D'Elia F Promoting children’s psychomotor development with multi-teaching didactics. Int J Environ Res Public Health. (2022) 19(17):10939. 10.3390/ijerph191710939 36078655 PMC9517746

[56] Rigon M Signorini G Scurati R Trecroci A Colella D Formenti D Multisport-integrated training for rugby instructors: success and effects on minirugby players. J Funct Morphol Kinesiol. (2024) 10(1):11. 10.3390/jfmk10010011 39846652 PMC11755658

[57] Parker M Curtner-Smith M. Preservice teachers’ use of production and reproduction teaching styles within multi-activity and sport education units. Eur Phys Educ Rev. (2012) 18(1):127–43. 10.1177/1356336/11430655

[58] Invernizzi PL Rigon M Signorini G Colella D Trecroci A Formenti D Effects of varied practice approach in physical education teaching on inhibitory control and reaction time in preadolescents. Sustainability. (2022) 14(11):6455–72. 10.3390/su14116455

[59] Bevans KB Fitzpatrick LA Sanchez BM Riley AW Forrest C. Physical education resources, class management, and student physical activity levels: a structure-process-outcome approach to evaluating physical education effectiveness. J Sch Health. (2010) 80(12):573–80. 10.1111/j.1746-1561.2010.00544.x 21087253 PMC3196855

[60] Chupina VA Pleshakova AY Konovalova ME. Methodological and pedagogical potential of reflection in development of contemporary didactics. Int J Environ Sci Educ. (2016) 11(14):6988–98.

[61] Taylor SL Noonan RJ Knowles ZR Owen MB Fairclough SJ. Process evaluation of a pilot multi-component physical activity intervention – active schools: Skelmersdale. BMC Public Health. (2018) 18(1):1383. 10.1186/s12889-018-6272-1 30563488 PMC6299621

[62] Bull FC Al-Ansari SS Biddle S Borodulin K Buman MP Cardon G World Health Organization 2020 guidelines on physical activity and sedentary behaviour. Br J Sports Med. (2020) 54(24):1451–62. 10.1136/bjsports-2020-102955 33239350 PMC7719906

[63] Piaget J. Structuralism. New York, NY: Basic Books (1970).

[64] Vygotsky LS. Mind in Society. Cambridge: Harvard University Press (1978).

[65] Fosnot CT Perry RS. Constructivism: a psychological theory of learning. In: Fosnot CT, editor. Constructivism: Theory, Perspectives, and Practice. New York: Teachers College Press (1996). p. 8–38.

[66] Rovegno I. The development of in-service teachers’ knowledge of a constructivist approach to physical education: teaching beyond activities. Res Q Exerc Sport. (1998) 69(2):147–62. 10.1080/02701367.1998.10607680 9635329

[67] Duncombe R Armour K. Collaborative professional learning: from theory to practice. J In-Serv Educ. (2004) 30(1):141–66. 10.1080/13674580400200230

[68] Bechtel PA O’Sullivan M. Enhancers and inhibitors of teacher change among secondary physical educators. J Teach Phys Educ. (2007) 26(3):221–35. 10.1123/jtpe.26.3.221

[69] Chow JY Davids K Button C Shuttleworth R Renshaw I Araújo D. The role of nonlinear pedagogy in physical education. Rev Educ Res. (2007) 77(3):251–78. 10.3102/003465430305615

[70] Rivoltella PC. Fare didattica con gli EAS: episodi di apprendimento situati. Brescia: La scuola SEI (2013).

[71] Bransford JD Stein BS Vye NJ Franks JJ Auble PM Mezynski KJ Differences in approaches to learning: an overview. J Exp Psychol Gen. (1982) 111(4):390–8. 10.1037/0096-3445.111.4.390 6219185

[72] Whitehead M. Definition of physical literacy and clarification of related issues. International Council of Sport Science and Physical Education (ICSSPE) Bulletin (2013).

[73] Edwards WH. Motor Learning and Control: From Theory to Practice. Belmont, CA: Cengage Learning (2010).

[74] Seifert L Smeeton N. A nonlinear pedagogy approach to promoting skill acquisition in young swimmers. In: Dekerle J, editor. High Performance Youth Swimming. New York: Routledge (2020). p. 200–12.

[75] Chamorro-Atalaya O Gonzáles-Pacheco A Quipuscoa-Silvestre M Durán-Herrera V Suarez-Bazalar R Vargas-Díaz A Professional and personal traits of the teacher and the relationship with didactic strategies. Int J Eval Res Educ. (2024) 13(1):122. 10.11591/ijere.v13i1.26957

[76] Bortoli L Robazza C. L’apprendimento delle abilità motorie. Due approcci tra confronto e integrazione, in: SDS-Rivista di cultura sportiva. Calzetti & Mariucci) (2016).

[77] Pesce C Croce R Ben-Soussan TD Vazou S McCullick B Tomporowski PD Variability of practice as an interface between motor and cognitive development. Int J Sport Exerc Psychol. (2016) 17(2):133–52. 10.1080/1612197x.2016.1223421

[78] Morano M Bortoli L Ruiz MC Campanozzi A Robazza C. Actual and perceived motor competence: are children accurate in their perceptions? PLoS One. (2020) 15(5):e0233190. 10.1371/journal.pone.0233190 32401796 PMC7219767

[79] Myer GD Kushner AM Faigenbaum AD Kiefer A Kashikar-Zuck S Clark JF. Training the developing brain, part I: cognitive developmental considerations for training youth. Curr Sports Med Rep. (2013) 12(5):304–10. 10.1097/01.Csmr.0000434106.12813.69 24030303

[80] Prain V Hickey C. Using discourse analysis to change physical education. Quest. (1995) 47(1):76–90. 10.1080/00336297.1995.10484146

[81] Wright J Forrest G. A social semiotic analysis of knowledge construction and games centred approaches to teaching. Phys Educ Sport Pedagogy. (2007) 12(3):273–87. 10.1080/17408980701610201

[82] Sebire SJ Jago R Fox KR Edwards MJ Thompson JL. Testing a self-determination theory model of children’s physical activity motivation: a cross-sectional study. Int J Behav Nutr Phys Act. (2013) 10(1):111. 10.1186/1479-5868-10-111 24067078 PMC3852537

[83] Ntoumanis N Quested E Reeve J Cheon SH. Need-supportive communication: implications for motivation in sport, exercise, and physical activity. In: Jackson B, Dimmock J, Compton J, editors. Persuasion and Communication in Sport, Exercise, and Physical Activity. Abingdon, UK: Routledge (2017). p. 155–69.

[84] Lee MCY Chow JY Button C Tan CWK. Nonlinear pedagogy and its role in encouraging twenty-first century competencies through physical education: a Singapore experience. Asia Pac J Educ. (2017) 37(4):483–99. 10.1080/02188791.2017.1386089

[85] Esposito G Ceruso R Aliberti S Raiola G. Ecological-dynamic approach vs. traditional prescriptive approach in improving technical skills of young soccer players. J Funct Morphol Kinesiol. (2024) 9(3):162. 10.3390/jfmk9030162 39311270 PMC11417948

[86] Bronfenbrenner U. The Ecology of Human Development. Cambridge, MA: Harvard University Press (1979).

[87] Cornelius-White J. Learner-centered teacher-student relationships are effective: a meta-analysis. Rev Educ Res. (2007) 77(1):113–43. 10.3102/003465430298563

[88] Raiola G D'Isanto T Di Domenico F D'Elia F. Effect of teaching methods on motor efficiency, perceptions and awareness in children. Int J Environ Res Public Health. (2022) 19(16):10287. 10.3390/ijerph191610287 36011930 PMC9408595

[89] Smeeton N Seifert L. Skill acquisition in the swimming pool. In: Dekerle J, editor. High Performance Youth Swimming. New York: Routledge (2020). p. 190–9.

[90] Montessori M. Il segreto dell’infanzia. Milano: Garzanti (2017).

[91] Guevara RM Moral-García JE Urchaga JD López-García S. Relevant factors in adolescent well-being: family and parental relationships. Int J Environ Res Public Health. (2021) 18(14):7666. 10.3390/ijerph18147666 34300116 PMC8306560

[92] Dobozy E. Constructivist and Montessorian perspectives on student autonomy and freedom. Proceedings Western Australian Institute for Educational Research Forum 1999. Kinestetik: Jurnal Ilmiah Pendidikan Jasmani (1999).

[93] Morrongiello BA Sandomierski M Schwebel DC Hagel B. Are parents just treading water? The impact of participation in swim lessons on parents’ judgments of children’s drowning risk, swimming ability, and supervision needs. Accid Anal Prev. (2013) 50:1169–75. 10.1016/j.aap.2012.09.008 23046692

[94] Chow JY. Nonlinear learning underpinning pedagogy: evidence, challenges, and implications. Quest. (2013) 65(4):469–84. 10.1080/00336297.2013.807746

[95] Moy B Renshaw I Davids K Brymer E. Preservice teachers implementing a nonlinear physical education pedagogy. Phys Educ Sport Pedagogy. (2019) 24(6):1–17. 10.1080/17408989.2019.1628934

